# Automatic detection of the autocorrelation-type measurement error component

**DOI:** 10.1155/S1463924686000068

**Published:** 1986

**Authors:** K. M. Hangos, J. L. Nagy, L. Leisztner

**Affiliations:** 1 Computer and Automation Institute Hungarian Academy of Sciences PO Box 63 Budapest H-1502 Hungary; 2 Institute of Forensic Science PO Box 314/4 Budapest H-1903 Hungary


Journal of Automatic Chemistry/*Journal of Clinical Laboratory Automation*, Volume 8, Number 1 (January–March 1986), pages 23–27

### Automatic detection of the autocorrelation-type measurement error component

**K. M. Hangos,**
*Computer and Automation Institute, Hungarian Academy of Sciences, H-1502 Budapest PO Box 63, Hungary*

**J. L. Nagy and L. Leisztner**
*Institute of Forensic Science, H-1903 Budapest PO Box 314/4, Hungary*

### Introduction

Automatic detection of measurement errors is extremely important in automatic analysis of large analytical sample sequences. Error detection usually involves samples from known control solutions which are regularly introduced into a sequence of unknown samples. The control can then be performed using proven graphical methods [1] based on the sequence of the measured control values during a longer period (day or shift). Numerical methods of detection are becoming increasingly important in microcomputer-based laboratory monitoring systems.

This paper discusses one component of the measurement error: the so-called 'autocorrelation-type' error. This error component appears when the measured values of subsequent samples influence each other; it is a frequent error in analytical measurements. Its main sources are either sorption phenomena or inertia effects in the measurement or registration devices.

The main purpose of this paper is to propose an efficient numerical method for the detection of autocorrelation-type measurement error components.

First, several models of the measurement error in automatic analysis are investigated. Based on this, the numerical method proposed is decribed. Finally, the results of the validation of the proposed method and its comparison with the graphical LAG-1 method is presented for control sample sequences.

### Materials and experimental methods

The sample sequences investigated were control samples with 0.5, 1.0 and 3.0 g/l Merck quality ethylalcohol concentrations. They were analysed with a Perkin-Elmer F42 gas chromatograph, which was equipped with an automatic headspace sampler. The sample sequences contained control samples introduced in a random manner in order to produce the autocorrelation-type error component. Flushing was used to remove the remaining part of the sample from the sampling capillary and LAB pipettes were used for sampling. The internal standard method was used for evaluation and a solution of 0.5 g/l l-propanol was added to each sample for this purpose. To avoid systematic error due to sampling, the internal standard was added to the sample with the same pipette and pipette-tip in all cases.

The sample sequences were repeatedly measured under different conditions. The parameters varied were sampling with the same or different pipettes and tips, and the duration of the flush.

Measurements were performed under the following conditions:

(1) *Sequence:* sampling with the same pipette (the first source of autocorrelation-type error) and with the same tip (the second source of autocorrelation-type error), 0 s flush (no flush) (the third source of autocorrelation type error);

(2) *Sequence:* sampling with the same pipette but three different tips, 0 s flush;

(3) *Sequence:* sampling with the same pipette and tip, 15 s flush (a weak source of autocorrelation-type error);

(4) *Sequence:* sampling with three different pipettes and three different tips, 15 s flush.

The evaluation of the chromatograms was performed by two separate methods: based on the ratios of peak areas or that of peak heights of ethylalcohol and the internal standard.

### The model of the measurement error sequence: the autocorrelation-type component

The sequence of the measured values can be denoted by:

$$\xi_t, \quad t = 1,2, \ldots N \qquad (1)$$

and the true values of the sample sequence by

$$X_t, \quad t = 1,2, \ldots, N \qquad (2)$$

for an automatically analysed measurement-sequence.

Their difference-sequence:

$$x_t = \xi_t - X_t, \quad t = 1,2, \ldots N \qquad (3)$$

can be called the 'measurement error sequence'. It is usually divided into a number of components (drift, random measurement error etc.), which have different physical and mathematical properties.

Several papers have been published recently on modelling measurement error processes in continuous-flow systems and in discrete samples. Wijtoliet [2] has thoroughly investigated measurement errors in gas chromatography, and several attempts have also been made to construct a generally valid measurement error process model [3 and 4].

The measured value sequence, as well as the measurement error sequence, can be modelled using discrete time stochastic processes (stochastic sequences). It can be shown that under relatively weak assumptions (assuming no discontinuity in the trajectories of the investigated processes with probability 1 and the Markov-property), the continuous time measurement error and measured value processes (in the case of continuously-flow samples) can be described by Ito-processes [5]. The discrete time model, i.e. the measurement error and measured value sequences can be derived from this model by applying equidistant sampling [6]. This discrete time model takes the form:

$$\xi_t = m(\xi_{t-1},t) + \delta\,(\xi_t, t)^*\varepsilon_t \qquad (4)$$

where

$m(.,.,.)$ a given deterministic function $\{m\ (\xi_{t-1},\xi_t,t) = \mu\ (\xi_t,t) + \xi_{t-1}$ and $\mu\ (\xi_t,t)$ the conditional mean function of the sequence $\xi_t$, $t = 1, \ldots, N\}$;

$\delta(.,.)$ a given deterministic function (conditional standard deviation function of the sequence $\xi_t$, $t = 1, \ldots, N$); and

$\varepsilon_t$, $t = 1,2, \ldots, N$ normally distributed white noise sequence with 0 mean and 1 variance.

In order to get simpler formulae functions $m(.,.,.)$ and $\sigma(.,.)$ in equation (4) are expanded in powers of $t$, and the terms of first order (for $\sigma$) and that of second order for m) are neglected. Thus the following equation is obtained from equation (4):

$$\xi_t = A(\xi_{t-1},\xi_t) + \mathrm{M}^*t + \mathrm{S}^*\varepsilon_t + X_t \qquad (5)$$

where

$A(.,.)$ is a given deterministic function { depending on $m(.,.,.)$ } ; and

M and S are constants.

There are three different terms on the right-hand side of equation (5) with different mathematical properties. The second and third terms do not depend on the previous samples, nor on the previous time; the first term depends on both the $t$th and on the previous sample. Thus the first term can be regarded as a general model of the autocorrelation-type error components. The second term is a fully deterministic linear function of time, so it can express a very simple linear drift. At the same time the third term is a fully stochastic, 0 mean white noise (with independent elements) sequence, which can be regarded as a random measurement error component.

The most simple linearized model of the measured value sequence can be obtained from equation (5) assuming the most simple linear form of the autocorrelation-type error component:

$$A(\xi_{t-1'},\xi_t) = a_0 + a^*(\xi_t - \xi_{t-1}). \qquad (6)$$

Substituting equation (6) into equation (5) gives:

$$\xi_t = a_0 + a^*\,(\xi_t - \xi_{t-1}) + \mathrm{M}^*t + \mathrm{S}^*\varepsilon_t + \mathrm{x}_t. \qquad (7)$$

Applying the definition of the measurement error sequence results in the following equation:

$$x_\mathrm{t} = a_0 + \mathrm{M}^*t + a(\xi_t - \xi_{t-1}) + \mathrm{S}^*\varepsilon_t. \qquad (8)$$

Note that in almost all practical cases, the standard deviation of the random measurement error component (S) and the mean of the measurement error ($a_0$ + M*$t$) depend on the true value $X_t$:

$$\mathrm{S} = \mathrm{S}(X_t) \text{ and } a_0 + \mathrm{M}^*t = A_0\,(X_t). \qquad (9)$$

### Detection of the autocorrelation-type error component

Detection requires testing the hypothesis on the presence of the autocorrelation-type measurement error component based on the mathematical model (equation (5)). The absence of the autocorrelation-type component is mathematically equivalent with the equality $a = 0$.

It can also be seen from equation (8) that the conditional probability distribution of the measurement error $x_t$, conditioned on the difference $(\xi_t - \xi_{t-1})$, is Gaussian with a mean (M*$t$ + $a_0$) and with a variance $\mathrm{S}^2$. So the following simple hypothesis can be investigated in practical cases instead of $a = 0$.

*Hypothesis 1:* the conditional mean and the conditional variance of the measurement error sequence conditioned on the difference $(\xi_t - \xi_{t-1})$ are independent of the condition.

In order to test this hypothesis, the domain of the possible values in the difference $(\xi_t - \xi_{t-1})$ must be divided by the points:

$$\xi_1 < \xi_2 < \ldots < \xi_K. \qquad (10)$$

With the help of these points, hypothesis 1, can be approximated by a set of other hypotheses as follows:

*Hypothesis 2:*

$$\left.\begin{array}{c} E[x_t|\rho_i \leqslant (\xi_t - \xi_{t-1}) < \rho_{i+1}] = E[x_t] \\ E[\mathrm{O}x_t^2|\ \rho_i \leqslant (\xi_t - \xi_{t-1}) < \rho_{i+1}] = E[x^2{}_t] \end{array}\right\} \qquad (11)$$
$$i = 1,2, \ldots, K\text{-}1$$

It is important to note that the set of hypothesis 2 is only an approximation of hypothesis 1, but it has a great practical 'advantage in this form'. As it can be seen from equation (11), the hypothesis can be easily tested with known statistical tests (F-test and t-test) and the necessary values in the condition can be easily computed from the measured value sequence, $\xi_t$, itself. The only problem from the computational viewpoint is how the values of the measurement error $x_t$ can be computed.

For this purpose, the control samples can be used because their true values, $x$, are assumed to be known. In this case it is assumed that the error of the control liquid sample preparation is negligible compared to the effects of the other measurement error components.

In the case of automatic analysis of large analytical sample sequences, control samples occur rarely in the sample sequences compared to the unknown samples. Thus the points in equation (10) must be chosen very carefully in order to have enough sample for testing each hypothesis in set 2 (equation (11)) for a good approximation of hypothesis 1.

In order to verify the numerical method, sample sequences containing only control samples were used in the authors experiments. This results in an increased number of suitable samples in the measurement error and in an obvious choice of the points in equation (10). The control samples with 0.5, 1.0, and 3.0 g/l ethylalcohol concentrations can produce differences ($\xi_t - \xi_{t-1}$) only in the region of the following values:

$$0.0, 0.5, -0.5, 2.0, -2.0, 2.5, -2.5$$

thus the values can be chosen as:

$\rho_1 = -3.0$; $\rho_2 = -2.25$; $\rho_3 = -1.5$; $\rho_4 = -0.25$; $\rho_5 = 0.25$; $\rho_6 = 1.5$; $\rho_7 = 2.25$; $\rho_8 = 3.0$.

### The proposed method and its application to control sample sequences

In order to show how tests for the set of hypotheses given in equation (11) can be performed easily, the computed quantities needed for the numerical method have been collected and arranged in tables 1–4, according to the four sample sequences described previously. The quantities in tables 1–4 have been computed from the measured values as follows.

A row in a table belongs to a given control sample concentration and to a given evaluation method (for example 0.5 g/l ethylalcohol concentration and ethylalcohol/internal standard peak area ratio evaluation).

The test of the hypothesis 2 set for each row is done in three steps:

(1) The number, the empirical mean and the empirical variance of the samples with the given concentration is computed and placed in the fourth column. After this, these samples are divided into three groups according to the previous sample concentration. The above characteristics (number, empirical mean and empirical variance) of each group is computed and put into columns one to three respectively.

(2) F-tests [7] can be used for testing hypothesis 2 (equation (11) for the variances. It is sufficient, however, to perform the test for the ratio of the columns with maximal and minimal variances. The computed F-value, together with the result of the hypothesis test, is put to the fifth column. The result of a test is positive ('+' sign) if the hypothesis has been proved true on the given significance level.

(3) If the result of the F-test is positive, the two sample t-tests can be applied to discover whether hypothesis 2 (equation [11]) holds for the mean values. In this case, it is also sufficient to perform the test for the columns with minimal and maximal means, applying the empirical variance of the fourth column as a common variance. The computed t-value and the result of the test can be found in the last column.

*Table 1. Data from measurement sequence 1.*

| Sample concentration (g/l) | | $X_{t-1} = 0{\cdot}5$ | $X_{t-1} = 1{\cdot}0$ | $X_{t-1} = 3{\cdot}0$ | Unconditioned | $F$ | $i/n$ | $t$ | $i/n$ |
|---|---|---|---|---|---|---|---|---|---|
| | | Peak height ratio of ethylalcohol/1-propanol | | | | | | | |
| 0·5 | $\bar{x}$ | 0·5166 | 0·5219 | 0·5190 | 0·5194 | 11·03 | | | |
| | $s_x^2$ | 1·1184E-4 | 1·2330E-3 | 4·0815E-4 | 5·9980E-4 | | | | |
| | $n$ | 12 | 15 | 14 | 41 | | – | | |
| 1·0 | $\bar{x}$ | 1·0157 | 1·0185 | 1·0008 | 1·0137 | 1·87 | | 0·7074 | |
| | $s_x^2$ | 1·9843E-3 | 2·9988E-3 | 3·5366E-3 | 2·5432E-3 | | | | |
| | $n$ | 18 | 14 | 8 | 40 | | + | | + |
| 3·0 | $\bar{x}$ | 3·0931 | 3·1218 | 3·1742 | 3·1236 | 1·72 | | 0·6772 | |
| | $s_x^2$ | 6·8099E-2 | 3·9616E-2 | 5·0129E-2 | 5·0220E-2 | | | | |
| | $n$ | 11 | 11 | 7 | 29 | | + | | + |
| | | Peak area ratio of ethylalcohol/1-propanol | | | | | | | |
| 0·5 | $\bar{x}$ | 0·5764 | 0·5737 | 0·5791 | 0·5763 | 1·31 | | 2·302 | |
| | $s_x^2$ | 4·4996E-5 | 4·4863E-5 | 3·4438E-5 | 4·4660E-5 | | | | |
| | $n$ | 12 | 15 | 14 | 41 | | + | | |
| 1·0 | $\bar{x}$ | 1·1369 | 1·1340 | 1·1405 | 1·1366 | 5·86 | | | |
| | $s_x^2$ | 9·7885E-4 | 1·6715E-4 | 9·0286E-4 | 6·4960E-4 | | | | |
| | $n$ | 18 | 14 | 8 | 40 | | – | | |
| 3·0 | $\bar{x}$ | 3·4202 | 3·4089 | 3·3993 | 3·4109 | 4·82 | | 0·8595 | |
| | $s_x^2$ | 4·2738E-3 | 8·8749E-4 | 2·2308E-3 | 2·3893E-3 | | | | |
| | $n$ | 11 | 11 | 7 | 29 | | + | | + |

*Table 2. Data from measurement sequence 2.*

| Sample concentration (g/l) | | $X_{t-1}$ = 0·5 | $X_{t-1}$ = 1·0 | $X_{t-1}$ = 3·0 | Unconditioned | F | i/n | t | i/n |
|---|---|---|---|---|---|---|---|---|---|
| | | Peak height ratio of ethylalcohol/1-propanol | | | | | | | |
| 0·5 | $\bar{x}$ | 0·5069 | 0·5161 | 0·5054 | 0·5095 | 5·44 | | | |
| | $s_x^2$ | 3·6267E-4 | 1·9730E-3 | 5·1038E-4 | 9·5145E-4 | | | | |
| | $n$ | 14 | 17 | 19 | 50 | | – | | |
| 1·0 | $\bar{x}$ | 1·036 | 1·0290 | 1·0143 | 1·0279 | 17·18 | | | |
| | $s_x^2$ | 1·2067E-3 | 3·0079E-4 | 5·1722E-3 | 1·8892E-3 | | | | |
| | $n$ | 19 | 18 | 13 | 50 | | – | | |
| 3·0 | $\bar{x}$ | 3·0605 | 3·0879 | 3·0443 | 3·0632 | 165·33 | | | |
| | $s_x^2$ | 2·1116E-2 | 1·2772E-4 | 1·2759E-2 | 1·1839E-2 | | | | |
| | $n$ | 18 | 15 | 17 | 50 | | – | | |
| | | Peak area ratio of ethylalcohol/1-propanol | | | | | | | |
| 0·5 | $\bar{x}$ | 0·5766 | 0·5972 | 0·5733 | 0·5823 | 17·48 | | | |
| | $s_x^2$ | 5·2546E-4 | 3·5678E-3 | 2·0410E-4 | 1·4971E-3 | | | | |
| | $n$ | 14 | 17 | 19 | 50 | | – | | |
| 1·0 | $\bar{x}$ | 1·1738 | 1·1541 | 1·1814 | 1·1687 | 4·20 | | | |
| | $s_x^2$ | 1·9391E-3 | 5·0186E-4 | 2·1103E-3 | 1·5333E-3 | | | | |
| | $n$ | 19 | 18 | 13 | 50 | | – | | |
| 3·0 | $\bar{x}$ | 3·4997 | 3·4887 | 3·4507 | 3·4797 | 1·65 | | 1·383 | |
| | $s_x^2$ | 1·3274E-2 | 8·3348E-3 | 8·5298E-3 | 1·0238E-2 | | | | |
| | $n$ | 18 | 15 | 17 | 50 | | + | | |

*Table 3. Data from measurement sequence 3.*

| Sample concentration (g/l) | | $X_{t-1}$ = 0·5 | $X_{t-1}$ = 1·0 | $X_{t-1}$ = 3·0 | Unconditioned | F | i/n | t | i/n |
|---|---|---|---|---|---|---|---|---|---|
| | | Peak height ratio of ethylalcohol/1-propanol | | | | | | | |
| 0·5 | $\bar{x}$ | 0·5194 | 0·5062 | 0·5194 | 0·5149 | 128·57 | | | |
| | $s_x^2$ | 1·6558E-4 | 1·3228E-3 | 1·0286E-5 | 5·1929E-4 | | | | |
| | $n$ | 14 | 17 | 19 | 50 | | – | | |
| 1·0 | $\bar{x}$ | 1·0540 | 1·0552 | 1·0447 | 1·0520 | 27·76 | | | |
| | $s_x^2$ | 1·4988E-3 | 6·9119E-4 | 5·3982E-5 | 8·2303E-4 | | | | |
| | $n$ | 19 | 18 | 13 | 50 | | – | | |
| 3·0 | $\bar{x}$ | 3·0809 | 3·1046 | 3·0820 | 3·0884 | 14·46 | | | |
| | $s_x^2$ | 4·1502E-4 | 6·0030E-3 | 1·4024E-3 | 2·4348E-3 | | | | |
| | $n$ | 18 | 15 | 17 | 50 | | – | | |
| | | Peak area ratio of ethylalcohol/1-propanol | | | | | | | |
| 0·5 | $\bar{x}$ | 0·6247 | 0·6234 | 0·6218 | 0·6231 | 1·26 | | 0·3824 | |
| | $s_x^2$ | 4·8197E-4 | 5·7022E-4 | 4·5042E-4 | 4·8094E-4 | | | | |
| | $n$ | 14 | 17 | 19 | 50 | | + | | + |
| 1·0 | $\bar{x}$ | 1·2551 | 1·2534 | 1·2856 | 1·2624 | 1·38 | | 2·196 | |
| | $s_x^2$ | 1·4420E-3 | 1·4008E-3 | 1·9363E-3 | 1·6830E-3 | | | | |
| | $n$ | 19 | 18 | 13 | 50 | | + | | |
| 3·0 | $\bar{x}$ | 3·7425 | 3·7303 | 3·7677 | 3·7474 | 1·34 | | 0·9106 | |
| | $s_x^2$ | 1·2728E-2 | 1·1374E-2 | 1·5252E-2 | 1·2883E-2 | | | | |
| | $n$ | 18 | 15 | 17 | 50 | | + | | + |

By devoting separate rows to each control sample's concentration, the dependence of the measurement error mean and variance (equation (9)) on the true value of the samples is taken into account.

As the result of the above procedure, there is no autocorrelation-type measurement error component under the given measurement circumstances and evaluation method if all the results of the corresponding F- and t-tests are positive on the given significance level.

It is important to note that the security of the autocorrelation-type measurement error component testing is influenced by the value of the $S^2(X_t)$ measurement error

*Table 4. Data from measurement sequence 4.*

| Sample concentration g/l | | $X_{t-1}$ = 0·5 | $X_{t-1}$ = 1·0 | $X_{t-1}$ = 3·0 | Unconditioned | $F$ | $i/n$ | $t$ | $i/n$ |
|---|---|---|---|---|---|---|---|---|---|
| | | Peak height ratio of ethylalcohol/1-propanol | | | | | | | |
| 0·5 | $\bar{x}$ | 0·5191 | 0·5177 | 0·5204 | 0·5185 | 3·23 | | | |
| | $s_x^2$ | 9·9173E-6 | 1·3014E-5 | 4·0266E-6 | 1·0522E-5 | | | | |
| | $n$ | 14 | 17 | 19 | 50 | | – | | |
| 1·0 | $\bar{x}$ | 1·0154 | 1·0171 | 1·0221 | 1·0177 | 3·92 | | | |
| | $s_x^2$ | 1·9756E-5 | 2·9915E-5 | 7·7638E-5 | 4·3323E-5 | | | | |
| | $n$ | 19 | 18 | 12 | 49 | | – | | |
| 3·0 | $\bar{x}$ | 3·0245 | 3·0287 | 3·0243 | 3·0256 | 1·07 | | 0·9329 | |
| | $s_x^2$ | 1·8883E-4 | 1·7665E-4 | 1·8120E-4 | 1·8385E-4 | | | | |
| | $n$ | 18 | 14 | 19 | 51 | | + | | + |
| | | Peak area ratio of ethylalcohol/1-propanol | | | | | | | |
| 0·5 | $\bar{x}$ | 0·6087 | 0·6167 | 0·6158 | 0·6141 | 1·17 | | 1·073 | |
| | $s_x^2$ | 3·8976E-4 | 4·5753E-4 | 4·0974E-4 | 4·1535E-4 | | | | |
| | $n$ | 14 | 17 | 19 | 50 | | + | | + |
| 1·0 | $\bar{x}$ | 1·2155 | 1·2097 | 1·2285 | 1·2166 | 1·69 | | 1·071 | |
| | $s_x^2$ | 2·0396E-3 | 2·6465E-3 | 1·5623E-3 | 2·1147E-3 | | | | |
| | $n$ | 19 | 18 | 12 | 49 | | + | | + |
| 3·0 | $\bar{x}$ | 3·6190 | 3·5948 | 3·6492 | 3·6236 | 1·89 | | 1·152 | |
| | $s_x^2$ | 1·0940E-2 | 1·4232E-2 | 2·0689E-2 | 1·5352E-2 | | | | |
| | $n$ | 18 | 14 | 19 | 51 | | + | | + |

variance. The smaller that variance, the smaller the effect that can be detected with a given sample size and with a given significance level.

### Conclusions

By applying this numerical and graphical (LAG-1) method for detecting autocorrelation-type measurement errors, it is evident that the results are the same for both methods.

In the case of the peak height ratio evaluation method, there were no such measurement circumstances when no autocorrelation-type measurement error component was present.

When applying the peak area ratio evaluation method, sequences three and four have been shown to have no autocorrelation-type measurement error component according to the numerical (see tables 3 and 4) and the graphical methods. This indicates that the circumstances of the flush have much more influence on the measurement error than do the other measurement circumstances (pipettes).

From the data of the numerical method (tables 1 and 2), it can also be seen that the empirical variances of the ratio of the peak height is much smaller than that of the peak areas. This fact is in good agreement with previous investigations [8]. As a consequence, the use of the peak height ratio allows the detection of smaller autocorrelation-type measurement error components than would be possible in the case of the ratio of the peak areas. At the same time, it can also be found that the empirical variance of the samples is much more influenced by the difference of the current and previous measured value, than the empirical mean of them, i.e. the autocorrelation-type measurement error component appears much more sensistive in the empirical variance.

The numerical method proposed here can be used for automatic validation and quality control of analytical measurements.

### References

1. Filliben, J. J., In *Validation of Measurement Process*, Ed. De Voe (ACS Symposium Series 63, American Chemical Society, Washington, D.C., 1977).
2. Wijtoliet, J. J. M., *Ultimate Retention Time Accuracy in Computer Assisted Chromatography* (Thesis, Eindhoven, 1972).
3. Currie, L. A., In *Treatise on Analytical Chemistry* Eds I. M. Kolthoff and P. J. Elving (Part 1, Vol. 1, 2nd edn, John Wiley and Sons, New York, 1978), 95.
4. Hangos, K. M., *Chemical Engineering Science*, **39** (1984), 1233.
5. Jazwinski, A. H., *Stochastic processes and Fitering Theory* (Academic Press, New York and London, 1970).
6. Hangos, K. M., *9th IFAC World Congress* **X** (Budapest, 1984), 39.
7. Rao, C. R., *Linear Statistical Inference and Its Applications* (2nd edn, John Wiley and Sons, New York, London, Sydney and Toronto, 1973).
8. Leisztner, L., Kuzmin, N. M. and Barna, P., *Zh Anal. Khim.*, **38** (1983), 2247.

